# Migrant and Refugee Impact on Well-Being in Rural Areas: Reframing Rural Development Challenges in Greece

**DOI:** 10.3389/fsoc.2021.592750

**Published:** 2021-03-11

**Authors:** Apostolos G. Papadopoulos, Loukia-Maria Fratsea

**Affiliations:** ^1^ National Centre for Social Research, Athens, Greece; ^2^ Harokopio University, Kallithea, Greece

**Keywords:** migrants, refugees, subjective well‐being, mobilities, migrant aspirations, rural Greece

## Abstract

The paper aims to exemplify and discuss the changing conditions and challenges posed by the newly arriving populations of migrants and refugees in rural Greece, along with local people’s views on the impact of the new arrivals in their rural places. Its main objective is to understand whether migrants and refugees create threats or opportunities for the local population, and whether movers and non-movers have a shared understanding of well-being in their rural areas. The analysis unveils the connections that are emerging between migrants and refugees and the economy, society and culture in rural receiving areas. Thus, the paper aims at showing the complexity of rural migrant flows and how the interactions between migrants, refugees and locals in the light of the well-being of rural areas may inform rural development in Greece. The paper is structured into five main sections following the introduction. The first section contains a discussion of the main concepts used as building blocks for creating a theoretical framing of well-being in rural areas. The second section develops a brief discussion of international and internal migration to rural areas in Greece, as well as providing some contextual information on the impact of the economic crisis and new developments in response to the recession. The third section includes a short presentation of the methodological approach and a description of the case study area. The fourth section is dedicated to an analysis of the narratives of international migrants, refugees, internal migrants, locals and stakeholders. Finally, the concluding section critically discusses the conceptualisations of rurality and well-being between the various population groups and articulates the challenges connected to well-being and mobilities in contemporary rural Greece.

## Introduction

It is argued that while economic migrants move between countries, and therefore places, in search of employment and/or a better way of life, refugees are forced to move away from their homes and end up in places where they have to settle and start their lives over. For a time, at least, after their initial movement(s), the two groups share a common objective: securing their living in a foreign place by adapting to the new setting(s) and pursuing a good life. In the relevant literature, migrating populations have another function in receiving countries and places: that of agents of social change (Castles 2004; O’Reilly 2012; Castles 2018); which is now widely recognised. The literature also notes that some moving populations, including potential movers, have a considerable degree of choice over where they move to (Bakewell, 2010), with the migration networks playing a facilitating role in this process (De Haas, 2010).

In such a context, migration is entangled with economic and social inequalities between sending and receiving countries, regions and places (Black et al., 2005). Moreover, permanent, temporary and circular movements of migrating populations are part and parcel of different and/or overlapping socio-spatial mobility patterns associated with movers. The socio-spatial characteristics of migrating populations need to be related to the scale and impact of migration. In this connection, the spatial perspective is important for analysing how and to what extent migrant and refugee impact on their places of settlement (Miller and Ponto, 2016). In an era of mobilities and identity politics, it is important to recognise the positioning of migrants and refugees in specific places and spaces (Zizek, 2016; Fukuyama, 2019). Their presence and settlement are negotiated at various spatial scales, while, at the same time, individual and subjective understandings are gaining ground in the discussions about the societal and democratic responses and moral obligations towards these populations.

In response to the common view of migrants and refugees as representing an external factor in local and rural places, we are now looking at both types of movers as populations who carry with them their imagined destinations and territorial imaginaries, which constitute important components of their mobility impetus. The “new mobility paradigm” (Hannam et al., 2006; Urry, 2007), to be discussed more extensively in the theoretical section, stresses the need to think of and analyse migration through the lens of movement, without neglecting sedentarism and anchored movements. Moreover, mobilities are complex assemblages of movements, social imaginaries and experiences (Glick Schiller and Salazar 2013; Salazar 2017). Analysing the mobilities puts us in a position to conceive and theorise the de-territorialisation and re-territorialisation processes in people’s lives.

In addition, migration is considered the complex result of an interplay between individual aspirations and contextual opportunities. Recent evidence suggests that aspirations are an important prerequisite for migration, but they are also reframed as a consequence of migration (Czaika and Vothknecht 2014; de Haas et al. 2019; Migali and Scipioni 2019). In many cases, migrants have much higher aspirations than non-migrants prior to migration, but also aspirations which are built up as a result of their migration experiences themselves (see also Creighton 2013; Carling and Collins 2018). Based on aspirations, the human well-being approach to migrants underlines the differences between subjective and objective well-being, emphasising the former (Wright, 2011).

From a rural perspective, there is a highly relevant discussion centered on those who move from urban to rural areas in search of a better—compared to the urban—way of life, aspiring to a “return to the land,” an “improvement of their well-being,” and/or a “rural idyll” (Halfacree, 2007). Terms such as “lifestyle migration” (Benson and O’Reilly, 2009) or “amenity migration” (Gosnell and Abrams, 2009) were coined to explain those who move to areas with rich natural resources and favourable climatic conditions, as well as to areas perceived as offering a better quality of life (Cadieux and Hurley, 2011; Matarrita-Cascante, 2017). Recent research has hightlighted the interaction between migrants (as newcomers) and rural places as important aspect of rural well-being (Gieling et al., 2017; Gieling et al., 2019). The relatively new term “rural cosmopolitanism” precisely captures how cosmopolitan dispositions and practices are connected to attributes of individuals and places in rural society (Cid Aguayo, 2008; Popke, 2011; Woods, 2018).

The paper aims to exemplify and discuss the changing conditions and challenges posed by the newly arriving populations of migrants and refugees in rural Greece, along with local people’s views on the impact of the new arrivals in their rural places. Its main objective is to understand whether migrants and refugees create threats or opportunities for the local population, and whether movers and non-movers have a shared understanding of well-being in their rural areas. The analysis unveils the connections that are emerging between migrants and refugees and the economy, society and culture in rural receiving areas. Thus, the paper aims at showing the complexity of rural migrant flows and how the interactions between migrants, refugees and locals in the light of the well-being of rural areas may inform rural development in Greece. Moreover, in the empirical analysis, we discern two notions of well-being: first, the “*well-being of rural areas*,” which relates to the general understanding of the qualities of living, working and/or residing in particular rural places, and second, “*well-being in rural areas*,” which refers to specific experiences of how migrants, refugees and/or locals describe their life qualities and their everyday practices in relation to local/rural well-being. In this context, the general understanding of the well-being of rural areas is juxtaposed against the specific conditions of personal well-being. A brief discussion of concepts such as mobilities, aspirations, subjective well-being, lifestyle and cosmopolitanism will be used to create a new theoretical context for reframing rural development challenges in Greece.

The paper is structured into five main sections: The first section contains a discussion of the main concepts used as building blocks for creating a theoretical framing of well-being in rural areas. The second section develops a brief discussion of international and internal migration to rural areas in Greece, as well as providing some contextual information on the impact of the economic crisis and new developments in response to the recession. The third section includes a short presentation of the methodological approach and a description of the case study area. The fourth section, based on empirical data collected in the period 2017–2020, is dedicated to the analysis of international migrants, refugees, internal migrants, locals and stakeholders’ perceptions and understandings of the well-being of rural areas and their own well-being as (permanent or non-permanent) residents in rural areas. The concluding section discusses the need for reframing rural development and articulates the main challenges connected to migration-and-development dynamics in rural Greece.

## Theoretical Framing for Understanding Migrant Well-Being in Rural Areas

The theoretical discussion in this paper draws on various disciplines: sociology, geography and their sub-disciplines, in the main, but also diverse research areas that seem to be converging in recent years. For example, it is now well-documented that internal and international migration should be seen as closely interconnected, both because they should be approached as different stages in migration processes, and to illustrate the many similarities in terms of concepts, mechanisms and trajectories in the two migration categories (Skeldon, 2006; King and Skeldon, 2010; de Haas et al., 2020). Moreover, the development effects of migration are discernible, when analysing the structural and functional aspects of migration, in the countries/regions/places of both origin and destination.

Some 20 years ago, Nyberg-Sørensen et al. (2002) were commissioned by the Danish Ministry of Foreign Affairs to study the existing and potential links between migration and development. The authors suggest that the positive dimensions and possibilities of the migration-development nexus should be taken into consideration, highlighting “[T]he links between migration, development, and conflict from the premise that to align policies on migration and development, migrant and refugee diasporas must be acknowledged as a development resource” (2002: 50). Such work had a great impact on related discussions, as well as coining the term “nexus” to illustrate the complex interlinkages between migration and development (Bastia and Skeldon, 2020). For example, Bastia (2018: 315), in her summing up of relevant attempts, refers to three phases of the migration-development nexus (which she also labels “optimist,” “pessimist” and “neo-optimist”) as follows: 1) during the 1950s and 1960s, when the focus was on remittances and return migration, and more specifically on filling labour gaps in the North; 2) during the 1970s and 1980s, when the focus was on underdevelopment and migration, with poverty and the brain drain as key themes; and 3) during the post-1990s period, when migration and co-development were stressed, transnational circulation celebrated, and temporary along with circular migration seen as the ideal.

The key question about who benefits from migration attached to the discussions on the “migration-development-nexus” cannot be answered on the basis of linear thinking (King and Collyer, 2016). In fact, there are various interpretations of this nexus, depending on an analysis of complex interdependencies, and on the two-way causality that may have paradoxical effects (King, 2018; Bastia and Skeldon, 2020). The dominant view, according to which migration is seen as a “tool,” which needs to be “properly” used to spread and/or promote development effects, depicts migration as a phenomenon collateral to development, as well as something that remains disembedded from the economic system. In this connection, it is considered of the utmost importance to get to grips with the social transformations along with the socioeconomic and political realities linked to migration (Castles, 2018; de Haas et al., 2020), but also to shift the discussion to the middle ground between migration and development as co-constitutive aspects of social reality (Raghuram, 2009; de Haas, 2020; Raghuram, 2020).

The human well-being approach offers interesting insights since it focuses on how well-being is constructed in specific places, the way(s) these constructions change during the migration process, and how well-being “travels” across spatial boundaries (Wright, 2012). This approach views migration as a strategy for exiting poverty and achieving well-being, reproducing many of the arguments underpinning the “migration-development-nexus,” but also considering both the positive and negative linkages between the two ends of the equation (Wright, 2011). However, the emphasis is on subjective well-being, along with life satisfaction and quality of life as these are perceived by migrants, while subjective well-being is de-aggregated into two components: the affective and the cognitive. Subjective well-being is built upon social comparisons (i.e. how migrants position themselves in relation to others) and needs that include everything needed to “live well,” while living well varies over time and people’s life course (Wright, 2012).

Additionally, the human well-being approach has important points of connection with the capabilities approach (Appadurai, 2004; Sen, 2007); but it remains plural and multiple. The capabilities approach underlines aspirations as an asset for migrants, but also cautions us that migration experience feeds back into and reinforces individual aspirations. However, aspirations and psychological characteristics are theorized primarily on an individual and/or intersubjective basis, while there have also been attempts to move towards relational well-being and retain the community or local level for conceptualizing migrant well-being (White, 2017). All in all, the subjective well-being approach is well connected to the discussion on migration and development in view of the “migration-development-nexus” and underlines the need to explore the nuts and bolts of such a nexus by exploring the complexity of related processes.

Such nexus is constructed locally, and it is thus important to remember that places are distinct mixtures of wider and more local social relations (Massey, 1991). Places should be understood with reference to peoples’ mobilities. It is important to think of places as nodes of social relations, which are continuously in the process of being reconstructed and negotiated in space. In Massey’s (Massey, 2005: 151) words we need to keep in mind the “throwntogetherness of places,” referring to the “even-shifting constellation of trajectories.” Places are negotiated by identities that are on the move, while there is multiplicity, antagonisms and contrasting temporalities. Places are practised by both movers and residents, and there is ongoing negotiation between intersecting trajectories. This produces a relational understanding of space which enables rural places to reconstitute, negotiate and hybridize (Woods 2007; Healey and Jones 2012).

In a similar vein, a long discussion on the population turnaround of rural areas, described as “counter-urbanisation,” depicts the diverse factors surrounding rural population growth and net migration to the detriment of larger urban centres; “counter-urbanization” remains a complex phenomenon challenged by various disagreements over its exact definition, rate and scope (Champion, 1998; Halfacree and Boyle, 1998; Mitchell, 2004). Due to rural migration, new social groups are emerging in rural areas, which are identified with diverse social and economic practices and engaged with innovative and/or hybrid activities, and whose lifestyles differ significantly from that of local people (Stockdale, 2006; Mahon, 2007; Stockdale, 2014a). Despite the complexity and indeterminacy of the term, the factors connected to counter-urbanisation are open to multiple interpretations, allowing for the rethinking of the facts behind the phenomenon (Argent, 2019). For example, counter-urbanisation may be the result of movements by former city dwellers who develop a genuine attachment to a rural place (or pro-rural counter-urbanisation see Halfacree and Rivera, 2012), or by urban dwellers who were driven away from the city by unemployment, the economic recession and so on. But there are also cases where there is multi-local living that primarily relates to better off socioeconomic groups. It is suggested that more targeted research is required to (re)connect mobilities, lifestyles and life-courses in rural areas (Argent, 2019: 762). Recent research has de-emphasized counter-urbanization as a comprehensive trend and sheds more light on the (inter)subjective and agency aspects of movements to/across rural areas (Stockdale, 2014b; Scott et al., 2017).

According to a recent review, “counter-urbanization” (Halfacree, 2012), “amenity migration” (Gosnell and Abrams, 2009), and “lifestyle migration” (Benson, 2010) are seen as analytical tools which ask and provide answers to different questions (Benson and O’Reilly, 2016: 23). Thus, whereas amenity migration and counter-urbanisation emerge out of geography and demography and focus on place and quantitative analysis, lifestyle migration leans towards qualitative analysis while also focusing on people and the identity-making projections of migrants. Lifestyle migration emerged two decades ago to theorize migration phenomena that could not be explained in terms of an economic rationale (O’Reilly, 2000). The basic idea behind lifestyle migration is that migrants create a narrative through which they render their lives meaningful (Benson and O’Reilly 2009). Lifestyle migration is therefore a complex and nuanced phenomenon that varies from one migrant to another and from one location to the next, while the category as a whole is difficult to operationalize (Benson and O’Reilly, 2016). Lifestyle looms large in this more recent discussion, playing an expanded role not only in terms of the style of life a migrant imagines in the new destination, but also in shaping the way of life actually lived after migration (Benson and Osbaldiston, 2014). Lifestyle migration has therefore developed an argumentation to suggest an upgraded role for lifestyle in migration (Benson and Osbaldiston, 2016; Benson and O’Reilly, 2016); the migrating imaginaries of a better life being the focus of the emphasis on lifestyle (Salazar, 2014).

This discussion of counter-urbanisation and lifestyle migration reflects both an understanding of changing rural space which articulates both structural and agency characteristics embodied in rural places (Halfacree, 2006; Woods, 2009), and a more individualistic understanding of rural space that focuses on personal attributes such as citizenship, emotions, everyday life and othering (Paniagua, 2016). Both lines of thought continue to contribute to the analysis of objective and subjective well-being in rural areas. However, it is important to keep in mind that migration and mobilities are core constituents of rural places (Halfacree, 2012).

The existence of various social actors in rural areas is tightly bound up with transformative mobilities--referring to a mixture of the numerous mobilities towards and among rural areas—which can be traced across rural and urban space (Papadopoulos and Fratsea 2020). The concept of mobility transcends the rural/urban dichotomy, since the rural is acknowledged as at least as mobile as the urban (Milbourne and Kitchen 2014). Along with mobilities which are central to the structuring of people’s lives, emphasis is also placed on (im)mobility, moorings, dwelling and stillness as well as on speed or liquidity (Bauman, 2007; Urry, 2007). The core contribution of the mobilities paradigm is that it focuses on the dynamics of movements without forgetting how peoples’ imaginaries and experiences anchor them to places (Faist, 2013; Sheller, 2014). Moreover, looking at mobility and immobility (sedentarism) on an equal basis as interconnected facets of social transformation (Glick Schiller and Salazar, 2013), permits immobility to be better explained and seen in conjunction with mobility (Schewel, 2019).

All in all, belonging emerges as a central notion in the discussion on migration/mobility and the construction of places. The sense of belonging includes two interconnected dimensions: “place-belongingness,” which refers to belonging as a personal, intimate feeling of being “at home,” and the “politics of belonging,” which refers to belonging in terms of claims and an official understanding of membership (Antonsich, 2010: 645). Therefore, place/regional attachment remains an important aspect of belonging in the current era (Antonsich and Holland, 2014). Countering the numerous attempts to relativise the “sense of belonging” and open up the boundaries of places to include migrants, strangers and cosmopolitans, defensive attempts have also been made to safeguard the integrity of the local, to praise parochialism, and stress the insideness of places (Tomaney, 2012; Tomaney, 2015). In the relevant literature, it is argued that the cosmopolitan-local divide is transformed into a cosmopolitan-local continuum, whereby various forms of attachment to local/national protectionism are identified (Roudometof, 2005; Olofsson and Ohman, 2007; Haller and Roudometof, 2010). This has given rise to novel understandings of citizenship and responsibility as these are promoted by cosmopolitanism in contradistinction from older understandings attached to localism/parochialism.

To sum up, own position is that rootedness (localness) and cosmopolitanism need to be reconciled and seen as co-producing aspects of places (Beck, 2002; Calhoun, 2002; Calhoun, 2008; Glick Schiller and Salazar, 2013). It is, thus, important to analyse the specific constellations of cosmopolitan/local attributes as these reflect on specific communities, places and regions. Place attachment relates to the well-being of both movers’ and non-movers’, and in fact enriches rural places (Berg, 2020). Both the reality and experiences of migrants and refugees, along with those of internal migrants (in counterurbanization and lifestyle migration terms), remain essential for co-constructing rural places and re-territorializing movers’ lives; both have an immense impact on rural development.

## Methodological Approach and the Region of Western Greece

This paper draws upon research implemented in the context of the IMAJINE project. Our research focuses on the multiple mobilities and their interconnections with actual and perceived social and spatial inequalities that are traced in both urban (the Attica Region and more particularly Athens) and rural areas in Greece (the Region of Western Greece). Between 2017 and 2020, qualitative semi-structured interviews were conducted with various (non)mover groups such as Romanian migrants, Syrian refugees, internal migrants into Western Greece and the local population. In addition, ethnographic observation in both research areas, interviews with stakeholders and key informants (i.e., policymakers, NGO representatives, farmers, and local authorities) at the national and local level, were used to triangulate information provided by the interviews. In total, 90 interviews were conducted over the two research areas, 59 of which were conducted in Western Greece (see Table 1). We acknowledge that the narratives of the interviewees are important sources of information and of emerging interpretation frames which are evaluated and (re)interpreted by the researchers. In this context, we take these narratives as respondents' “truth claims,” but we have also tried to juxtapose these various truth claims and triangulate with information collected from other sources to illustrate the underlying processes.

**TABLE 1:** Interviews conducted in Western Greece.

Population group	Male	Female
Stakeholder/key informants	9	2
Local population	8	5
Syrian refugees	10	5
Romanian migrants	5	8
Internal migrants	1	6
***Total***	33	26

Source: Fieldwork 2017–2020.

In our opinion, it is important to hold a critical realist stance when dealing with different data sources, while also reveal our reasoning (Given, 2008: 226–230; Bakewell, 2010; Iosifides, 2012). The qualitative research design allowed us to glimpse respondents’ understanding and actions and, in addition, to assemble these glimpses to make sense of diversity, convergence and/or divergence in relation to well-being. In this way, the various actors interact within a common socioeconomic setting, contributing significantly to the (re)construction of the specific places and attach meaning to shared notions and aspirations motivating their practices. The rich empirical material offers elements of subjective and shared understandings of well-being and therefore enabled our informed interpretation of migrant and refugee impact on rural well-being.

The Region of Western Greece has been the focus of our research for over a decade. Our long-term presence in the area has been important in allowing us to monitor and critically evaluate socioeconomic developments alongside wider policy and economic developments at the national level. But entering and accessing the research field is crucial in any research, and particularly when participants belong to vulnerable groups such as migrants and refugees (Neuman, 2014). Although we were “outsiders” or “strangers” asking “strange” questions (Neal and Walters, 2006), our familiarity with the wider area and the fact that our research team combined a range of personal characteristics in terms of gender, languages spoken, age group and rural/urban background^1^ helped create an environment of trust and build rapport with the participants. It is argued that this kind of collaborative model combines the advantages of insiders’ ease of access with the outsiders’ fresh perspective, particularly when conducting multi-sited research involving multiple languages (Fitzgerald, 2006).

In more detail, the population groups were located through migrant and civil society organizations, personal contacts, and snowball sampling. Care was taken to include participants who combined different social and demographic profiles (i.e. gender, educational level, family status, stage in the life-cycle, length of residence in the country/area etc.). Questions addressed to the interviewees covered their migration/mobility history and aspirations and their perceptions and experiences of the effect of migration on the area and material and non-material aspects of their well-being and future plans. Migration research poses particular and important challenges when conducting cross-language and multiple language research (Squires, 2006; Inhetveen, 2012). Interviews were carried out in Greek, Romanian, English, and Arabic—as members of the research team spoke those languages—, and when possible were recorded with the participants’ permission; alternatively, extensive notes were kept. With the exception of the interviews conducted in Arabic, which were translated into English, coding and analysis took place in the language in which the interview took place. We conducted thematic analysis on the data, following Charmaz’s (2006) “flexible” grounded theory approach to data coding and analysis. The analysis that follows focuses on the approaches of non-movers, residents (locals) living in rural areas in Western Greece, regarding the contribution of the various categories of movers against the movers’ own assessment of their well-being.

In fact, the wider area of Western Greece has experienced various population movements (e.g., in-migration and out-migration and/or seasonal movements) in different periods. These included movements of international migrants who had been living and working in different urban or rural regions of Greece and saw new employment prospects in the intensive agricultural sector, but also internal migrants originating from large urban centres who settled in the area, combining employment opportunities with the search for a better quality of life. Based on the Population Census data, almost one third have moved to the research sites from another rural or urban centre, particularly from Athens, while this tendency has increased in pace over the last 20 years or so. These developments were combined with the out-migration of locals towards urban centres for educational and employment purposes, leading to the depopulation of the most marginal villages. More recently, several Syrian refugees have been living in the Myrsini open refugee camp while they wait for their asylum claim to be accepted and/or recognised under the refugee regime. In short, the area includes places that have experienced both depopulation and internal and international migration.

Our research site comprises two Regional Units in the Peloponnese peninsula--Ilia and Achaia—which belong administratively to the Region of Western Greece. The plains of the Regional Unit of Ilia in Western Greece are the largest in the Peloponnese, but the region is also known for the coastal wetlands of Kotichi and Kaïafa, which are areas of rare natural beauty and ecological value. In terms of economic activity, agriculture, stock breeding and food processing have long been the main economic activities of the local population, alongside tourism. Currently, agriculture is still an important pillar of the economy of Ilia (27.3% of employment), although most of the population is employed in the service sector (48.9%). The Regional Unit of Achaia has a limited primary sector (8.1%) and an adequately developed tertiary sector (66.5%) (ELSTAT, 2011). There has been an expansion in horticulture and greenhouse cultivation, while more recently strawberry growing has experienced a rapid rise; currently, over 90 percent of Greece's strawberries are grown in Western Greece, and specifically in the villages of Manolada and Nea Manolada (Papadopoulos and Fratsea, 2017). The capital of Achaia is Patras, which is the third largest city in Greece and Greece’s main port to Italy.

International migration to the area dates back to the early 1990s, when it was primarily connected with the collapse of the socialist regimes in neighbouring Balkan countries. Currently, Albanians comprise the majority of the migrant population, followed by Bulgarian, Romanian and Asian migrants (see Table 2). Following the recent refugee/migration crisis in 2015, a refugee camp was established in Myrsini Village, in a former holiday resort called “LM Village”, in 2016. This initiative was also facilitated at the time by the mayor of the area, who is of Syrian descent. Currently, 280–300 Syrian refugees are living in this small camp.

**TABLE 2:** Migrant population of Regional Units of Ilia and Achaia.

Country/Region of origin	Achaia	Ilia	Total
EU	3,130	4,638	7,768
* Romania*	*808*	*950*	*1,758*
Other European	11,919	7,687	19,606
* Albania*	*11,149*	*7,379*	*18,528*
Africa	578	103	681
Asia	1,370	2,128	3,498
Other countries	442	333	775
***Total***	17,439	14,889	32,328

Source: ELSTAT, Population Census 2011.

The Region of Western Greece is not considered homogeneous, but rather a multiplicity of social spaces that overlap in the same geographical area (Cloke and Milbourne, 1992). The area comprises small villages, towns, coastal areas, environmentally-protected areas and remote places, all of which are affected by various forms of mobility. Hence, fieldwork was conducted in various localities within the Regional Units, including different areas such as Valtholomio, Myrsini, Arkoudi, Amaliada, Aghios Nikolaos, Manolada, Lechaina and Lapa. All these areas can be considered mostly agricultural, with some having a “mildly” touristic profile.

## Intertwined Stories of Mobility and Well-Being in Western Greece

The determinants of quality of life in the Greek countryside are discussed in the interviews with the various population groups. Generally, the accounts offered here underscore the marine and natural environment, place attachment and a sense of belonging, how stress-free, serene and calm life is compared to life in the city, but also the lack of accessibility and connectivity with other areas and the limited infrastructures and implications of the prolonged economic crisis. What is more, which factor prevails in the narrative of each population group differs depending on age, life phase, marital status, gender and occupation.

The analysis of the qualitative material reveals at least two discourses: definitions and aspects of the *well-being of rural areas* and individual perceptions and practices of *well-being in rural areas*. The former relates to the characteristics of the rural areas themselves as they are assessed by the different population groups. These characteristics include the natural environment, local infrastructures, and the implications of different migration flows in the area, while the latter refers to subjective interpretations of well-being which are linked to emotions, aspirations, hopes, dreams, imaginations, and a sense of belonging to the rural community. Mobility lies at the heart of well-being, but the relationship between mobility and well-being is complex and multifaceted and differs between population groups. By and large, residents consider mobility/migration essential in economic and demographic terms for the enhancement of the well-being of rural areas, while movers see it as a strategy for advancing the quality of life of individuals who move to the countryside and for improving one’s own well-being in those places. As we shall see in the analysis of the interviews, the relationship between perceptions of well-being of and within rural areas is not straightforward; rather, conflicting interests, views and discourses are foregrounded by the different population groups.

### Aspects of Well-Being in Rural Western Greece

The beauty of the natural environment is one of the themes that figure strongly in people’s narratives explaining the quality of life in the area. In general, interviewees underlined the unique characteristics of “their” countryside, which combines forests and agricultural landscapes with the marine environments of the coastal communities. Examining the narratives of the locals, one identifies an unspoken pride connected to their place of residence, its characteristics compare to other rural or urban areas, and what it offers. They often underscore the role of the village’s marine environment for the quality of life of the place. As Maria argues:

*“[O]ur sea is very nice. If we take care of it [our sea] (…) I criticize anyone who does not take the care needed to keep our beach clean. We have a beautiful sea, not deep, in which anyone can swim, even by crawling”* (Maria, 64 years old).

Interestingly, the role the natural environment plays in well-being in rural areas is more often underlined by those who recently moved to such places. For many, this combination of marine and agricultural landscape acted as a pull factor in the deliberation process leading up to their decision to migrate. Many Romanian migrants include the natural environment among the factors that influenced their decision to stay in Western Greece. As Dorina explains, Western Greece is a place that combines all the characteristics of a natural and marine environment. As she says: “*I like it here, because here [in the plain] I have the sea. There, there’s the mountain. I have everything here”* (Dorina 50 years old). Along similar lines, a “better” life closer to nature is considered a “family dream,” a goal achieved by moving to another place. Anton explains that the initial hardships of moving to another country are “worth it,” since he can provide his family with a better quality of life in the coastal village: “*There are many [factors] I consider important in my decision to stay here. [My son] says to me, "Dad, when will we go to the sea?.” We will not go this week, but we will go next week. I put €10–20 gas in the car and off we go to the sea. And what a sea! If we were in Romania, "When will we go to the sea, dad?" would mean me having to work for a year to travel 600 km by car and spend 1,000€ for a week for the three of us (…) the Black Sea is nice, but not for me* (Anton 37 years old). Such narratives show that beyond individual interpretations of well-being there is a family perspective to well-being where individual and family quality of life intersects, and personal quality of life is assessed in view of the well-being of the members of the family.

As expected, in the aftermath of an extremely severe economic recession, assessments of well-being in rural areas hinge equally on economic factors. Although the impact of the crisis varied between sectors and geographical areas (Papadopoulos et al., 2019), the ramifications of the crisis for the local economy were given particular emphasis during the interviews. Two opposing discourses surface from the analysis of qualitative material: On the one hand, the contraction of the national economy resulted both in rising unemployment/underemployment, but also in a cutting back on consumption in the local economy. Tasia (59 years old), a shop owner in a small village, remembers with nostalgia the “previous” years before the financial crisis, when—she claims—consumption was higher: “*The economic situation is tough in the village. I'm on the verge of closing the store.*” In a similar vein, Angelos emphatically illustrates the implications of the crisis. He considers the economic situation in the region to have deteriorated since 2009: “*Year by year, the situation has worsened. In fact, it’s been a drama here this year [2019]. No one comes into the store and, generally speaking, the shops aren’t doing any business anymore. Consumers are turning to the big supermarkets, which are cheaper, and letting the smaller shops close. Here, our local market [in the central square] was full of shops. Now ... everything is closed” *(Angelos 70 years old). Yet, for those working in the primary sector, agriculture remained a buffer for securing some income. In fact, as Nikos argues, those who worked in agriculture were more resilient during the recession: *“The financial crisis is… a special case. Somehow, we [the farmers] have always been in “crisis.” We have always been living through a crisis, you know… I remember always putting something aside for a rainy day (…) I looked ahead. We don’t own extensive land property, instead we have 200 olive trees and a small plot in the village* (Nikos 65 years old).

Interestingly, the newcomers in the wider area, Romanian migrants and internal migrants alike, acknowledge the severe implications of the economic recession for the Greek economy, contest the local entrepreneurs’ view of limited opportunities and consider rural areas to be places of opportunity rather than scarcity. The well-being of rural areas is anticipated and considered better compared to the cities. They have a romanticised view of their current place of residence, which they see as a destination characterised by a growing number of employment positions, a place where investments are achievable, and a new future lies ahead.

As the following quote reveals, the interviewee moved to the local village when she found a permanent position as a civil servant there. As the economic environment was rather insecure, she left her job in Athens. In her opinion: “*There are professional opportunities here. Only that. To tell the truth, in the private sector you simply have no idea how long [you’ll have a job]. There’s an expiration date. For a woman, the private sector is very hard.”* (Xanthi 45 years old). In the same vein, another interviewee worked for many years in Athens and abroad as a film and art director, but by 2012 couldn’t get any new projects. She therefore decided to follow her husband to his ancestral village, where he owned some land with a house and a small farm. She moved to a village for the first time in her life. She made a new start as a housewife, a small food producer and a shop owner, while she also made certain steps to promote culture and art in the area. She describes her position like this: *“[I] came here and became an economic migrant”* (Violetta 52 years old).

Yet, for the Syrian refugees living in the area, well-being is not closely associated with labour market opportunities. The majority of pathways to labour market participation are precarious and unstable jobs. In fact, there are numerous quotes describing problems they face finding paid employment in the local labour market. We observe different attitudes on the part of respondents in relation to their integration into the local labour market, while the exploitation they experienced in Turkey (prior to coming to Greece) often continues once they are in Greece.

*“I tried in Kylene [a nearby town]. I go to Kylene almost every day, going there. Talking to people, trying to find job, work, not lucky. Someone offered work (…) olive trees. I worked about 20 days, and he gave no money. I pay tomorrow, I pay tomorrow, 20 days and he disappeared*. (Mohammed, 30 years old).

*“At first, we were working in a greenhouse. They still haven’t paid us.”* (Shana, 32 years old).

*“[I found work] at the greenhouses last summer, and I was working for a farmer. He didn’t give us water. [I was paid] 15 euro every day. [For work of] Nine hours*. (Usama, 25 years old).

Most interviewees agree that mobilities have had a positive impact on the well-being of rural areas, although each population group foregrounds different aspects of migration/mobility. It became obvious during the interviews that some rural villages have been losing population for a long time. The young generation did not really aspire to staying and living in these areas but wanted to move to nearby cities (such as Patras), Athens or even abroad instead. In this sense, these areas have been experiencing a process of depopulation and are mostly inhabited by older people. This feeling of depopulation or abandonment came over strongly in local people’s narratives. This is confirmed by the last Population Census which shows that the wider area’s population declined by 7.6%, in spite of the recent migration flows. Many respondents underlined the need for investment and “better” infrastructure to attract younger people back to their localities, illustrating the contestations of the local development dynamics.

“*This place needs youth and jobs and then everything else will take its course. The place does not need anything else* (Foteini, 47 years old).

*“In the area I would like to have two playgrounds. I cannot go because I am getting older, but for the children who will come here, to have [as a place] something to make them happy, something good. Because I pass through some villages where there are like not even 10 people, 15 people, they are families. (…). This is what I would like in the village. (…). Not costly infrastructure, just two squares to hang out in. That would be very nice. Let the people gather… That is, to do something, to attract the people [back]. That’s what I think is missing”* (Maria 64 years old).

In general, in-migration is considered beneficial for the well-being of rural areas. However, most of the local population distinguished between the various forms of migration. Over the years, due to the different population movements and migration flows, a rural ethnic diversity has emerged. Attitudes and perceptions regarding migration vary, depending on ethnic background and length of residence in the area. More to the point, Albanians, Romanians and Bulgarians who migrated to the area in the 1990s are generally seen as part of the local economy and society; they live in the villages with their families, work there for years and years, and their children eventually go to school with the children of local people. A more “utilitarian” approach is taken with the more recently arrived Bangladeshis and Pakistanis who work in the intensive agriculture. These nationalities are mainly represented by single men, with limited participation in the local public sphere. Although their contribution to the local agricultural sector is frequently voiced, their presence in everyday life is generally silenced. The presence of asylum seekers in the nearby camp facility of Myrsini, on the other hand, is acknowledged but their presence in the area is considered provisional.

*“[There are migrants in the area] I see some people who have a darker skin. I think they are from India or somewhere. I know them; I saw them last year, too. Of course, there are also some Albanians, they have been settled here for many years; they have their houses here. They have their families. Everything is ok, it’s all good. (…) [they] shop here. They speak broken English. I think they work locally in agriculture. They do not cause any problems”* (Afroditi, 32 years old).

By the same token, Tasia argues that migration is crucial for the regeneration of rural areas: “*Migrants who have families, they have not changed the place. They have changed the place positively. They work here. They help the economy. ...But also, the other people from Pakistan, Bangladesh they also help, too. They come, shop, and spend their money. And they are well-behaved people”* (Tasia 59 years old).

In economic terms, immigration contributed to the survival and expansion of the agricultural sector in the area. Many interviewees said that that the younger generation was reluctant to work in agriculture, and migrants thus help grease the wheels of the local agricultural labour market. *“The young people would not do the kind of jobs we have here that need to be done [agricultural jobs]. They would not work in the fields. Do you think the Greeks would do agricultural work? How do you see it? I do not see Greek people working in the fields, just Albanians and other nationalities...To be honest, we get migrants to do our jobs, too”* (Afroditi 32 years old). This quote confirms the prevalent trend of migrants replacing the indigenous labour force in agricultural activities in Greece and across southern Europe (Papadopoulos 2015; Corrado et al. 2016).

A few interviewees expressed negative opinions about the effects of migration on rural well-being. For example, one respondent said that migration has not really helped the area, arguing that migration was having a generally negative impact. This negative impact was allegedly connected to the presence of migrants, which creates conflicts in the community:

*“Migration creates frictions here; it creates social frictions. In general, it creates a lot of talk between people here... the local people do not go out much, they do their shopping and go back to their houses. I think migration has played a role in that. I believe that migration has played some sort of role in that”* (Markos 39 years old).

Apart from these positive or negative representations of migration, there were many informants who held contradictory opinions and stances on migration and its effects on the area. These perceptions would simultaneously communicate both positive and negative aspects of the effects of migration on the local environment. For instance, Aris, a retiree who spent most of his life in Athens and only returned to his village after he stopped work, argued that migration is beneficial for the area, as it provided much-needed labour. However, he also stated migration has changed the place for the worse. His rationale for doing so was based either on cultural reasons or issues relating to law and order (Aris, 73 years old).

To sum up, the contribution of mobilities to the well-being of rural areas is a rather vexed issue. The vast majority of the interviewees referred to the positive impact on the local demography and economy, as migrants cover labour shortages in some economic sectors and support local consumption. Most of the internal migrants, viewing their current rural area of residence with a cosmopolitan gaze, tend to express more positive attitudes about international migrants and refugees. The negative implications relate more to the allegedly ethnic antagonisms between migrants which have the potential to hinder social cohesion. Therefore, although many locals acknowledged the fact that the economic crisis impacted on migrants as well as locals, some locals expressed the opinion that the severe economic crisis might have changed the way in which local people perceived migration. This supports findings from public opinion polls conducted during the period of the economic crisis. According to this line of thought, the economic crisis has created a less hospitable environment for migrants and has negatively influenced local perceptions of migration.

### Everyday Practices of Well-Being in Rural Areas

The narratives of movers and non-movers alike make it clear that well-being in rural areas is closely related with their hopes, dreams and aspirations, leading to various constructions of the “rural.” In this context, the “good life” in rural areas is associated with emotions, feelings, and social relations. The “rural life” is imagined and experienced differently by the various categories within the different population groups, while their perceptions of living well in rural areas change during the life-course. Hence, the meaning of what is a “good” or “bad” rural life may change for local residents, internal migrants, international migrants and refugees.

Locals relate their individual well-being in rural areas to the rural place itself. In their description, they say they could not imagine themselves living somewhere else or having the good life in another place. Being born and raised there, they feel rooted and have deep ties to the land and to other members of the community. Looking back in retrospect, Tasia argues: *“Our [family] life in the village was so good…that I don’t know if anyone else has ever had such a good life, whose life unfolded like ours”* (Tasia 59 years old). Similarly, many locals argue that the good life consists not only of material aspects, but also far more importantly of the interpersonal relationships that develop in a place, of sociability and belonging. As Costas argues, “*I have never thought about leaving this place, I feel I belong here*” (Costas 45 years old). Angelos argues more emphatically: “*I was born here, I was raised here, and I will die here. I do not see any reason to go anywhere else (…) life here is not like in Athens or big cities where you don't know your neighbour. Here everybody knows everybody”* (Angelos 70 years old).

For migrants and refugees alike, initial aspirations play an important role in the decision to migrate: they plan their life in a place other than their current residence based on the imaginaries of potential and/or real destinations. For most Romanian migrants, imagining a “good life” somewhere else was part of their social imaginaries even before the collapse of the socialist regime (Fratsea, 2019; Fratsea and Papadopoulos, 2020). Migration seemed like a potential means of starting anew after a watershed event in their life, and/or like a strategy to improve their well-being. In this context, social networks help them construct “images” about places and facilitates the creation of a vision of what life may be like there. The story of Nora (33 years old) is telling. She explains how her family members, who lived in Western Greece, would send her pictures and paintings of landscapes and vineyards in the Peloponnese. Later in life, when she had the opportunity to move to another country, she recalls: *“I wanted to go to Greece!”*. Although many factors contributed to her decision to move to Greece, she argues that those images of a different life in the countryside stayed with her until her arrival.

In a similar vein, Constantin’s brother was already in Greece before Constantin embarked on his migration journey. As he recalls: “*In my mind, I imagined Greece like heaven, you know… Summer vacations, pleasant climate (...) OK, I didn't have a specific picture, no detailed picture about exactly how life was here…But life here is different [compared to how he imagined it]. Not that it disappointed me, on the contrary, I understood that to live well you must work, nothing is taken for granted*” (Constantin 37 years old). Constantin associated his initial aspirations with specific feelings invoked by images of the natural beauty of rural Greece. As he had just finished high school at that time, the destination area was associated with feelings of freedom and joy, of being carefree. Positive or difficult experiences that arise later on de-romanticize these initial—youthful—aspirations, and rural areas became places where someone can increase their well-being and/or improve their social status.

For Syrian refugees, on the other hand, living well is feasible anywhere but their country of residence; in a place where they feel secure and respected (Papadopoulos and Fratsea 2019). More often than not, they do not relate initial aspirations to specific places, but rather to specific countries in which they believe they would have a better standard of living. Germany, Belgium and other countries in Western Europe are among their desired destinations. Yet, they implicitly compare the livelihood they experience in the villages of Western Greece with the life they could have in the big cities of Western Europe. They construct a specific picture of what life chances they would have there out of information gleaned from networks, the media and social media.

The following quote captures their initial aspirations before leaving their country, as they describe “where” the good life can be found, where he envisions his family growing up: [in] *“a country that understands my value. The country that appreciates my work, my labour. The one that respects my humanity. And which helps me? This is my country. Not the country that kills me and kills my family, and throws me in jail, and tortures me. That country is not my home. You know what I mean? I mean, I am being very honest. I mean, just waking up each morning, having a coffee with my wife, going out with her to buy some stuff, and I say to her, “How can I not love Greece?.” We talk about it, my wife and I, and we say that, if only there were more job opportunities… otherwise, I would never leave Greece”* (Makram 36 years old).

The majority of Syrians refugees say they feel “at home” in Greece. The notion of “home” includes various configurations and meanings (Ralph and Staeheli, 2011). Among the factors they mention is remembrance, the strong similarities between Greece and Syria. It seems they perceive various aspects such as the landscape, the geomorphology, the environmental setting, peoples’ attitudes and cultural attributes as being similar between the two countries. According to their interviews, all these factors can contribute to a better life and to their developing a sense of belonging and place attachment. However, they mainly see their presence in the area as temporary; they adopt a kind of “tourist gaze” (Urry, 2002) as they wait for the travel documents (“Ausweis”) to move to another country. For instance, Sara, who lives temporarily in the camp in Myrsini village waiting for her asylum application to be approved, recalls living on the islands when she first arrived in Greece, and says that living close to nature is better compared to other places. She explains how she and her sister made small trips and visited the Neda river in the Western Peloponnese, a place of natural beauty and attraction. After the interview, she showed us pictures of all the places in the Peloponnese and Zante island that she had visited.

For Romanian migrants, feeling “at home” and a sense of belonging depend on the social relations one develops in the place of residence over the life course: *“You get attached to the place…you know, for the last few years, when I’ve gone back to Romania, on the way back, when I cross through the Greek-Bulgarian border, I say ‘I’m home’…and when I get from Patras to Pyrgos and then onto the narrow country roads, I say to myself “Ah, I’m finally home”* (Aurelia, 49 **years old).**

When examining internal migration towards Western Greece, at least two types of internal migrants emerge (cf. Halfacree, 1995): first, those you were born and grew up in Western Greece, but moved away for educational or employment reasons later in their adult lives, and second, those who were born and raised elsewhere (e.g., Athens), with no prior connections to the area, but who decided to live in Western Greece. Examining internal migrants’ narratives, we can discern two constructions of well-being in rural areas, which are illustrated in the following examples:

Foteini was born in a village in Western Greece 47 years ago. She moved to Athens for studies and stayed there for 25 years, working as an accountant. Although she never imagined herself living in a rural area, she saw a better life for her children there. As she says: *“You are next to nature. (…) The reason I had no problem returning [here] and knowing that I would not have people to socialize with, was because I knew I could do what I’m doing today—I’ll take the car and in 5* *minutes, I can be at the sea or in the forest, I can take the dog or my child, or go alone, stay for an hour, walk, run, collect shells [on the beach] and that covers my needs. Okay, I'm older now, too. If I was 20* *years old, this wouldn't cover my needs. It didn’t back then, and that’s why I left and never came back” (*Foteini, 47 years old). Life in rural Greece is connected to a better, more relaxed and healthier quality of life compared to the city. Yet, it is evident that the stage in the life cycle plays an important role. Put differently, the perception of the rural place differs depending on the stage in the life course. As Foteini says, she feels that she appreciates the quiet life and the slower rhythms in the countryside more, now that she is older, while her children have a better life living in an environment close to nature compared to life in cities where pollution is higher. Hence, the well-being of the family in the rural area is better compared to the city.

Niki, 45 years old, was born in a village in Western Greece. Initially, she moved to Athens to attend university and then moved to Western Europe where she started her family and established a business. However, she explains that a few years ago: *“I didn't have any free time. I was feeling that the more I engaged with more, the more I wanted to advance my work, but I had no time for anything else and I wanted to enjoy my family life, my children, a little. (…) Yes, I was in this situation and I wanted to see what else there is in life! I remember saying to myself: ‘Do I want to be like this forever?’” *(Niki 45 years old). Eventually, she and her husband decided to move back to her place of origin. She explains their rationale: *“It was a call we’d discussed for some time. (…) Many times, we used to say 'How nice, it would be for the kids to go out in the fields and play, to be outside in the countryside, to have the rest of the family close by, their grandmother and their grandfather. My husband’s family are scattered around other places. (…) We said we’d try another way of life, a more humane way of living! And we finally made the decision—we left and came to Greece. (…) I confess that it was a shock, because I’d been there for nearly 20* *years, and we were used to another way of life”*. In other words, her decision to move back to rural Greece was based on the imagined quality of life that she and her family would enjoy there, but also on the possibility of strengthening relations with her family.

Yet, constructing rural places from far away can result in idealized views of well-being. As Niki confessed later in her interview, there is some distance between the expected well-being and the reality. A sense of belonging and place attachment does not always follow on from prior memories of place and growing up at home: *“All those years [of absence], when you visit the place, you are on vacation, and of course everything is nicer when you are on vacation.” *In fact, many internal migrants confess that at times they feel like “strangers” in the place they have settled in. Sometimes, they felt socially excluded and able to communicate only with other internal migrants, with whom they share a code of “communication.” Thus, they sometimes feel detached from the rural places they initially belonged to. Foteini feels dislocated and explains: *“You feel…stiff…you are a stranger… When I am at home, in my space, on my beaches and in my fields, I feel at home. But when I am with the people who live here, I feel like a stranger. In objective terms, I was a stranger because I was in Athens for 26* *years. The people I remembered living here have passed away. I do not recognize anyone from the younger generation, because people my age have left the village. Therefore, I am a stranger” *(Foteini, 47 years old). The analysis of how internal migrants see their living in the area of their residence illustrates their sense of cosmopolitanism, which does not compromise easily with local attitudes and manners. Evidently, many of them may feel closer to the international migrants and refugees than to the locals; however, the local people are more accepting of internal migrants than international migrants and refugees.

## Conclusion

This is the first time research has been conducted in Greece into how the four population groups—i.e. international migrants, refugees, internal migrants and the locals—perceive their well-being in rural areas and their well-being in relation to the other groups. The discussion of the qualitative findings reveals the complexity of well-being in rural Greece, especially when the perceptions and attitudes of the various population groups are analysed in detail. Some of this evidence confirms previous research on the strategic role of migrant labour in Greek agriculture (Papadopoulos, 2015; Papadopoulos and Fratsea, 2017), while other reflects public discourses during and in the aftermath of the economic crisis.

The “locals,” who are identified here with the non-movers, tend to have a more naturalistic and traditionalist view of well-being in rural areas associated with “parochialism” and pride in living in the same settlement all their lives. Some locals may originate from nearby villages or from mountainous areas, and thus consider themselves as originating from the wider area, but the movers consider them all to be equally local. The scalar conceptualisation of the “locals” and internal migrants reflects an inability to demarcate between locals and cosmopolitans, since in real life the local/rural well-being merges various groups together.

The internal migrants share certain views with the international migrants as regards their understanding of rural areas as places of opportunity, since they acknowledge the existence of employment positions in agriculture and tourism as well as in self-employment. This view contradicts locals’ perception of rural areas as being in decline, due to the low availability of economic opportunities. Moreover, a segment of the internal migrants shares a touristic gaze with refugees who see their rural area of residence as attractive due to its natural amenities and environmental qualities; at the same time, however, they recognise the lack of infrastructure and services which could further improve their material well-being. In their turn the locals acknowledge their sense of belonging and stress that they feel “at home.”

The existing asymmetry between subjective and objective well-being in Greek rural areas, which is bypassed by the locals who assign more emphasis to their emotions and feelings about their place of residence, is acknowledged by all the other groups, who realize that the material well-being should be also prioritized alongside the subjective well-being. However, there is a distinction between two main groups: 1) those—the internal migrants and international migrants--who are keen to stay in rural areas, to be connected to rural areas and, at the same time, to try and build on the existing employment opportunities and/or undertake reasonable entrepreneurial initiatives; and 2) those who feel like sojourners—the refugees--, since they have no attachments to the rural places due to the lack of networks and/or initiatives on the part of the receiving society, but also due to the “imaginaries” of those movers in search of “greener pastures” in other countries.

Interestingly the “rural imaginary” becomes important among those who already feel that they are socially and economically integrated into local/rural places, but not among those whom the receiving rural places consider to be in “transit,” “unconnected,” or “exotic.” The mechanisms of rural well-being include socio-economic parameters alongside belonging and place attachment.

All in all, two major intertwined challenges seem to emerge for well-being in rural areas in Greece: first, the challenge of improving the material well-being in rural areas by strengthening and supporting the role of internal and international migrants, who have positive aspirations and are in a position to take actions that will help regenerate their places economically; and second, the challenge of creating incentives and empowering newcomers as agents of change that will benefit rural places, and especially those places which are experiencing depopulation and decline. To address both challenges, the power geometry in rural places needs to be rebalanced in favour of the most dynamic and resourceful elements within them; in other words, to enable the newly-arriving populations to imagine themselves living in rural places.

## Data Availability

The datasets presented in this article are not readily available because they are confidential as agreed upon with the study participants. Requests to access the datasets should be directed to the corresponding author.

## Footnotes

1 One should take into account that, thanks to historical and structural factors, there are still strong bonds between urban and rural areas in the south of Europe. Unlike in the majority of northern European countries, Greek socio-economic development was not accompanied by a wide disengagement from the rural (Papadopoulos 2018). What this means for the interviews with the ‘local’ population is that referencing this “rural” origin made it easy to initiate the interview and enhance the trust between the participants.
